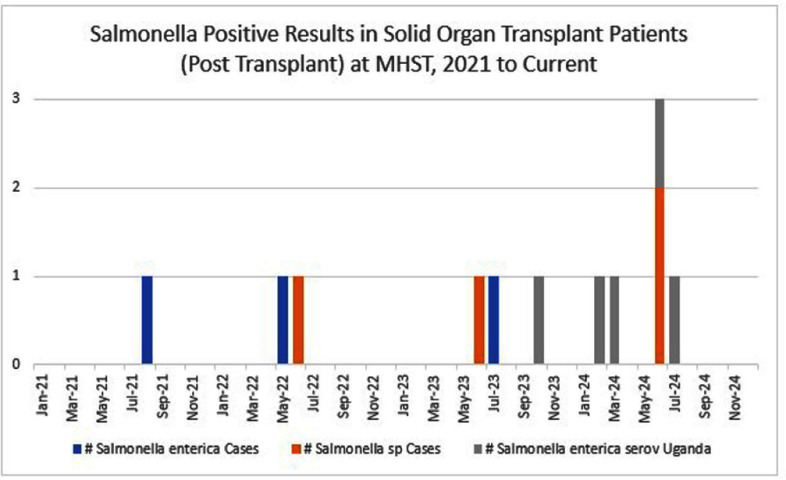# Cluster of healthcare-associated Salmonella enterica serotype uganda in solid organ transplant patients

**DOI:** 10.1017/ash.2025.371

**Published:** 2025-09-24

**Authors:** Claire Jai, Rosemary Garcia, Victoria Davis, Jeffrey Jones, Kristen Beals, Julia Moody, Ken Sands

**Affiliations:** 1 Methodist Healthcare System and HCA; 2 Methodist Specialty and Transplant Hospital; 3 HCA/Methodist; 4 San Antonio ID Consultants; 5 Methodist Hospital Specialty and Transplant; 6 HCA Healthcare

## Abstract

**Background:** Between October 2023 and July 2024, Methodist Hospital Specialty Transplant (MHST, San Antonio TX) experienced a cluster of five infections with Salmonella enterica serotype uganda among hospitalized kidney and liver transplant patients. All patients were symptomatic and specimens included blood, stool and urine. Salmonella enterica serotype uganda is rare with only a few reported outbreaks in the literature with none from healthcare settings. **Methods:** Investigation focused on two hypotheses: 1) a source within the facility was causing broad exposure but only severely immunocompromised patients were becoming symptomatic or 2) The clinical management of certain transplant patients is creating a risk of reactivation of Salmonella sp. The case definition was any solid organ transplant patient with a positive culture result (any specimen source) for Salmonella enterica serotype uganda post-transplant (no defined time) with or without symptoms who had a hospitalization at MHST after October 2023. The response focused on horizontal control measures (foundational infection prevention practices, water management), vertical control measures (food and nutrition services, patient screening) and epidemiologic descriptive analysis. **Findings:** Whole genome sequencing identified the five cases to be from an identical species. Cases occurred among kidney and liver transplant patients in roughly the proportion to the underlying census of these patients. No clinical or nutritional product or service was identified that would expose risk to transplant patients exclusively. There were no commonalities among the cases in relation to clinical care, procedures, or type of immunosuppression. Screening was performed for twenty-eight patients (either pre-liver transplant, post liver transplant or post kidney transplant) hospitalized between September and October 2024 with either chronic diarrhea or acute loose stools, none of which were positive for any species of Salmonella. Inspections of the kitchen showed opportunities around staff attire, food handling, food storage and the physical environment. Compliance to infection prevention assessments was 71% initially but improved to above 90% by early October 2024. Hand hygiene by food handlers after the handling of raw meat was low in July and August 2024 at 25% and 37%, respectively. After targeted interventions, compliance increased to 80% by September 2024. **Conclusion:** Following interventions, no additional healthcare-associated Salmonella sp cases in transplant patients were noted after July 2024. While a common source for these cases was suspected, none was definitely identified. While it was not possible to make a definitive conclusion, evidence suggests that the transplant population had a unique vulnerability to this species of Salmonella.

**Figure:**